# Endoscopic Endonasal Intraconal Approach for Orbital Tumor Resection: Case Series and Systematic Review

**DOI:** 10.3389/fonc.2021.780551

**Published:** 2022-01-03

**Authors:** Xin Zhang, Wei Hua, Kai Quan, Guo Yu, Zunguo Du, Zixiao Yang, Xiaowen Wang, Jianping Song, Liang Chen, Wei Zhu

**Affiliations:** ^1^ Department of Neurosurgery, Huashan Hospital, Shanghai Medical College, Fudan University, Shanghai, China; ^2^ National Center for Neurological Disorders, Shanghai, China; ^3^ Shanghai Key Laboratory of Brain Function and Restoration and Neural Regeneration, Shanghai, China; ^4^ Neurosurgical Institute of Fudan University, Shanghai, China; ^5^ Shanghai Clinical Medical Center of Neurosurgery, Shanghai, China; ^6^ Research Unit of New Technologies of Micro-Endoscopy Combination in Skull Base Surgery (2018RU008), Chinese Academy of Medical Science, Shanghai, China; ^7^ Department of Pathology, Huashan Hospital, Fudan University, Shanghai, China

**Keywords:** orbital tumor, intraconal, nasal-cranial base tumor, neuroendoscope, endonasal approach

## Abstract

Intraorbital tumor could be approached by numerous surgical methods. The neuroendoscopic endonasal approach could provide a feasible corridor for indicated tumors. Herein we present a series of 6 consecutive intraorbital tumors from April 2018 to October 2020, which received endonasal endoscopic resection. Cadaveric dissection was performed for the intraconal approach, and the literature was also reviewed. Five tumors were located intraconally, while one extraconally. The pathology revealed 1 angioleiomyoma, 1 cavernous hemangioma, 1 pilocytic astrocytoma, 1 meningioma, and 2 schwannomas. Five of the six achieved gross total resection, including 3 tumors with lateral extension beyond the optic nerve. Preoperative visual deterioration was observed in 4 of the 6 patients, and all got improvement postoperatively. Transient oculomotor nerve palsy was presented in one patient postoperatively. No cerebrospinal fluid leakage, enophthalmos, or strabismus was observed. The median follow-up time is 27 months (11~41 months). At the 6-month follow-up, the visual acuity remained unchanged compared with that at discharge. Proptosis was resolved in 2 of the 3 patients; diplopia was improved in one patient. In conclusion, endoscopic endonasal intraconal approach could be suitable for selected pathological conditions, and for both medial or beyond medial extraconal and intraconal orbital tumors.

## 1 Introduction

The intraorbital tumor is a variety of conditions commonly seen by neurosurgeons. The most common symptoms are exophthalmos and visual deterioration (1). The surgery is challenging for both neurosurgeons and ophthalmologists because of the deep-seated position and adherence with the cranial nerves. A variety of surgical approaches have been used in the management of the tumors involving the orbit. External approaches to the orbit have been well established, including transconjunctival, transcutaneous, and transcranial approaches (2, 3). External orbitotomies are usually performed with or without osteotomy. Norris et al. applied the endoscope to remove orbital foreign bodies and treat fistula through transconjunctival or external approaches in 1981 (3). An orbit-zygomatic approach could be applied for large tumors involving the lateral orbit or for the exposure of the optic canal.

The endoscopic endonasal approach could provide a minimally invasive corridor to the skull base, extending from the cribriform plate to C2. The lateral skull base tumor involving the orbit and the pterygopalatine fossa could also be approached *via* an endoscopic endonasal approach, pioneered by Kassam AB et al. (4, 5). Herman first described the endoscopic endonasal approach for the removal of an orbital cavernoma in 1999 (6). The endoscopic endonasal approach could provide an alternative corridor for the intraorbital lesions. Several studies demonstrated that this approach was suitable for the inferomedial orbital tumors, especially soft benign extraconal tumors (2, 5, 7). Ma et al. reported 23 intraorbital cavernous hemangiomas (CHs) transnasally resected with endoscopy in a single institution (8). Paluzzi summarized an algorithm of “round-the-clock” surgical access to the orbit, in order to guide surgeons to choose the most appropriate approach (9).

Herein, we present a consecutive cohort of 6 intraorbital tumors with different pathologies in our center to illustrate our opinion on choosing the best approach. The clinical features, surgical outcomes, and technical advantages are presented, and related literature is reviewed.

## 2 Patients and Methods

### 2.1 Patient Population

A retrospective study of 6 consecutive cases of intraorbital tumors from April 2018 to October 2020 was conducted, all of which were surgically resected *via* a purely endonasal endoscopic approach in the Department of Neurosurgery, Huashan Hospital, Fudan University. Medical records and radiological images were reviewed. Surgical approaches, complications, cranial nerve outcomes, and follow-up were analyzed. All the patients were fully informed with written consent after approval by the Huashan Hospital Institutional Review Board.

### 2.2 Anatomic Study

We performed an anatomic study in the laboratory. The endoscopic endonasal intraconal approach was applied on two cadaveric heads. Intraorbital neurovascular structures from the endonasal view were dissected and exposed.

### 2.3 Surgical Technique

The patient was placed in the supine position with the head slightly rotated toward right and the chin tilted toward the surgeon. Magnetic resonance image (MRI)- guided neuro-navigation was applied during the operation. Abdominal incision was prepped for later skull base reconstruction in case of cerebrospinal fluid (CSF) leakage. A four-hand bi-nostril technique was applied with a 0° or 30° endoscope (Karl Storz, Germany), and the ipsilateral middle turbinate was resected to expand the surgical corridor. After removal of the lamina papyracea, the periorbita was exposed. The optic canal bony decompression was performed for intraconal tumor. The intraconal surgical corridor was created between the medial and inferior rectus muscles. After removal of the tumor, the medial orbital wall was reconstructed with Gelfoam and bone fragment and fixed with BioGlue. A nasoseptal flap was harvested in circumstance of grade 2 or grade 3 CSF leakage (10).

### 2.4 Postoperative Examination and Follow-up

After the surgery, prophylactic antibiotics were prescribed for 48 h, and the lumbar drainage was applied under the circumstance of intraoperative CSF leakage. The visual acuity and visual field were examined by the ophthalmologists and neurosurgeons postoperatively, and the head MRI was performed before discharge for most cases. The patients were scheduled with a follow-up MRI 3 months after the surgery. Surgical complications were identified through the operative reports and postoperative clinic notes.

### 2.5 Literature Search

A literature search was completed in September 2021 using the PubMed database. We used a search strategy for (“transnasal” or “transsphenoid*” or “endonasal”) AND (“endoscop*”) AND (orbital disease [MeSH Terms]) AND (“tumor*” OR “hemangioma*” OR “schwannoma*” OR “meningioma*” OR “glioma”). Non-English articles were excluded.

### 2.6 Statistical Analysis

Statistical analyses were performed using SPSS (version 20). All statistical methods were two-tailed test, and a p value of < 0.05 was considered significant.

## 3 Results

### 3.1 Clinical Features

The demographics and clinical features of the six patients are shown in **Table 1**. The mean age of the four patients is 37 years, including five female and one male. Unilateral visual loss is presented in four patients, with the worst of no light perception. Proptosis is presented in three patients and diplopia in one patient (**Table 1**).

**Table 1:** Characteristics of the demographics and surgical outcomes.

No.	Age (years), sex	Side	Symptoms	Pre-op CN deficits	Location/relationship with CN II	Pathology	EOR	Post-op CN palsy	Adjuvant therapy	Follow-up
1	8, F	Rt	Heterotropia	II (NLP)	Intraconal/Md, Ltl	Pilocytic astrocytoma	GTR	II unchanged	None	No recurrence
2	41, M	Lt	Proptosis	/	Intraconal/Md, Ltl	Schwannoma	GTR	Transient III palsy, proptosis resolved	None	No recurrence
3	46, F	Rt	Proptosis, diplopia	VI	Extraconal/Md	Angioleiomyoma	GTR	Proptosis and VI palsy resolved	None	CR
4	43, F	Rt	Visual loss, proptosis	II	Intraconal/Md, Ltl	Meningioma	PR	II and proptosis unchanged	None	SD
5	34, F	Rt	Visual loss	II	Intraconal/Md	Schwannoma	GTR	II unchanged	None	No recurrence
6	47, F	Lt	Visual loss	II	Extraconal/Ltl	Hemangioma	GTR	II unchanged	None	No recurrence

EOR, extent of resection; F, female; M, male; Rt, right; Lt, left; NLP, no light perception; Md, medial; Ltl, lateral; GTR, gross total resection; PR, partial resection; CR, complete remission; SD, stable.

The median maximal diameter of the tumor is 27.9 mm (range, 20.0–47.8 mm). The pathology includes 1 angioleiomyoma, 1 cavernous hemangioma, 1 meningioma, 1 pilocytic astrocytoma, and 2 schwannomas. Regarding the tumor location, four are intraconal and two are extraconal. Four of the six tumors invaded lateral to the optic nerve, and the other two tumors confined inferiomedially. Three of the four tumors with lateral occupation were intraconally located.

The age prevalence of intraorbital lesions is unremarkable according to previous reviews (11). The patients usually present with proptosis, visual impairment, local pain, and diplopia (1). The most seen pathologies of intraorbital tumors are slow-growing benign tumors, such as hemangioma, schwannoma, optic nerve sheath meningioma (ONSM) and osteomas.

### 3.2 Adequate Opening and Optic Decompression Could Be Achieved *via* the Endonasal Intraconal Approach

**Figure 1:**
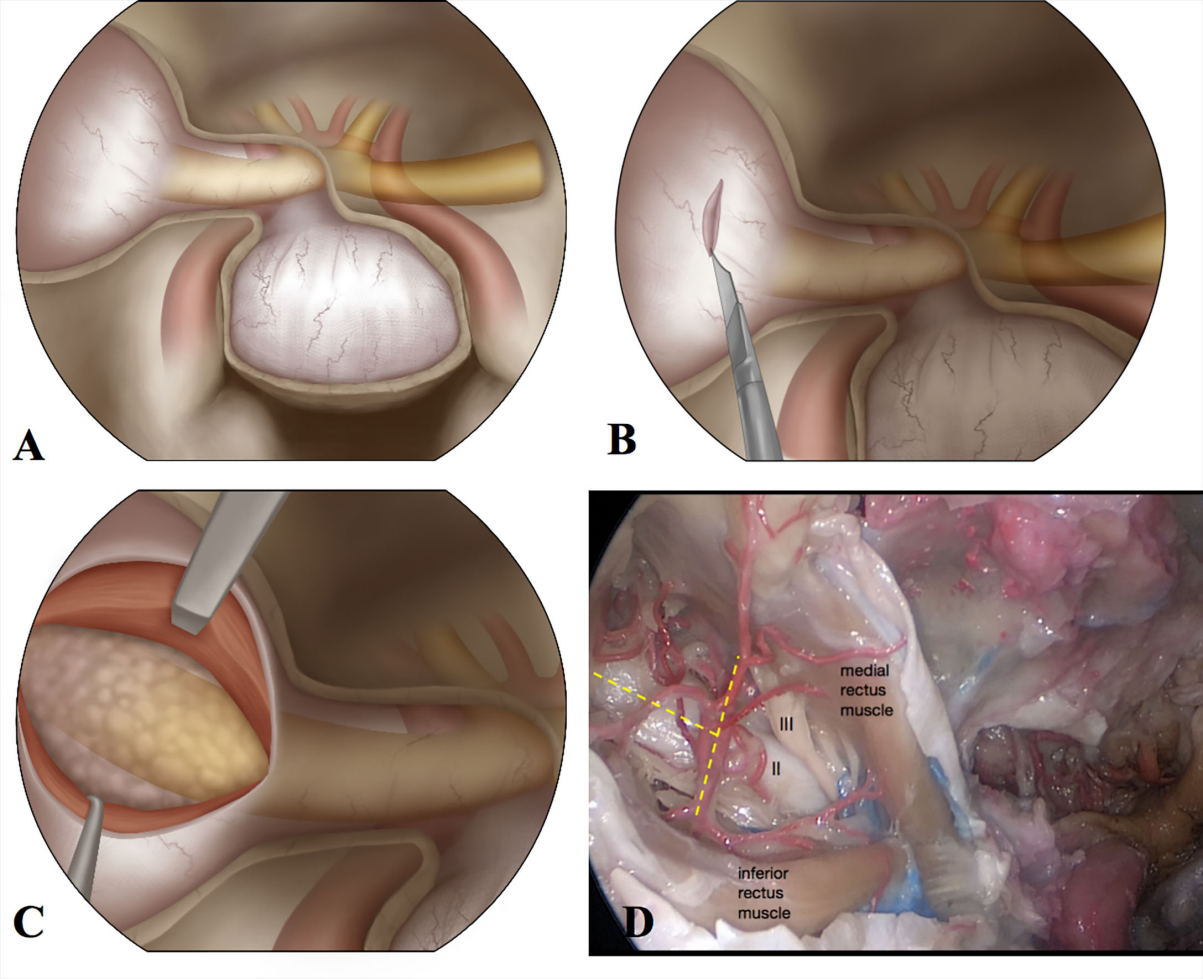
The illustration of surgical steps and anatomic dissection of the orbit from an endoscopic endonasal view. **(A)** After opening the sella floor, lamina papyracea, and optic canal, right orbit is exposed. **(B)** The periorbita is cut open with a blade. **(C)** Retracting the medial rectus muscle and inferior rectus muscle to expose the intraconal lesion. **(D)** The anatomic dissection of the orbit from the endoscopic endonasal approach. The optic nerve, oculomotor nerve, and branches of ophthalmic artery are exposed.

In this consecutive cohort, all 6 cases received middle turbinectomy to expand the corridor to the medial orbit. Four patients with intraconal tumor received optic canal bony decompression. Surgical technique and steps are shown in **Figures 1A**–**C** (see *Patients and Methods*). Intraoperative CSF leakage occurred in one patient with pilocytic astrocytoma. Five of the six cases achieved gross total resection, including three tumors with lateral invasion to the optic nerve. Only one case with intraconal meningioma got partial resection and optic canal bony decompression, considering extreme adherence with the optic nerve and copious bleeding. Previous literature on surgical resection of intraorbital tumors is reviewed in **Table 2**.

**Table 2:** Literature review of the intraorbital tumors using purely endoscopic transnasal approach.

Year/author	Cases	Location	Approach	Outcome
2004, Tsirbas	3	Orbital apex	Combined transnasal and transconjunctival	NA
2010, McKinney	6	Intraconal	Transnasal	67% GTR
2012, Castelnuovo	16	Intraconal medially located	Transnasal	8 GTR extra-intraconal/6 biopsy intraconal, diplopia
2013, Muscatello	3	Inferio-medial orbit	2 transnasal, 1 external	All GTR
2014, Chhabra	5	Medial orbital		4 GTR, 1 STR, transient diplopia, enophthalmos
2014, Healy DY	1	Intraconal	Transnasal	GTR
2014, Karligkiotis A	3	Extraconal involving medial orbital wall	Transnasal	Resolution of ophthalmological symptoms
2015, Arai Y	4	Extraconal or intraconal/medial or lateral	2 transnasal/2 staged surgery(craniotomic/transantral)	2 cases endo biopsy first then transfer to craniotomy/transantral
2015, Shin M	15	Aggressive tumor involving the orbit	Transnasal	12/15 GTR
2016, Shafi F	1	Intraconal orbital apex	Transnasal	Biopsy
2016, Chen YB	11	Optic canal	Transnasal	All GTR, visual all improved
2017, Sun MT	2	Medial orbital apex	Transnasal	GTR
2018, Montano N	70	Orbital	Craniotomic/transnasal/trans eyelid	28 ONSM, 14 CH, 6 schwannoma
2019, Castelnuovo	2	Intraconal, inferomedial	Transnasal	GTR, complete resolution of symptoms
2019, Ma JY	23	7 extraconal, 16 intraconal	Transnasal	16 of 23 GTR

GTR, gross total resection; NA, not available; ONSM, optic nerve sheath meningioma; STR, subtotal resection.

We performed orbital dissection of the endoscopic endonasal intraconal approach on two cadaveric heads. After removing the lamina papyracea and the medial wall of the orbit, we created the corridor between the medial and inferior rectus muscles. The optic nerve, oculomotor nerve to the medial rectus muscle, and branches of the ophthalmic artery could be exposed well from this approach (**Figure 1D**).

### 3.3 Surgical Outcome

Pre- and postoperative neurological functions of the six patients are shown in **Table 1**. Cranial nerve (CN) II impairment in patients 1, 4, 5, and 6 remained stable postoperatively, without newly developed visual impairment. Transient CN III palsy was presented in patient 2 postoperatively, which was resolved at the 6-month follow-up. Preoperative CN VI palsy of patient 3 was resolved 6 months after the surgery. Two of the 3 patients with proptosis recovered postoperatively. No patient complained of CSF leakage postoperatively. No enophthalmos or strabismus was observed after tumor resection.

### 3.4 Long-Term Outcome

By September 2021, the median follow-up time is 27 months (11~41 months). At the 6-month follow-up, the visual acuity remained unchanged compared with that at discharge. Proptosis was resolved in 3 patients; diplopia was improved in patient 3. No patient received chemotherapy or radiotherapy after the surgery.

### 3.5 Case Illustration

Two cases are illustrated below, and the other four cases are shown in the **Supplementary Materials**.

#### 3.5.1 Case 1

**Figure 2:**
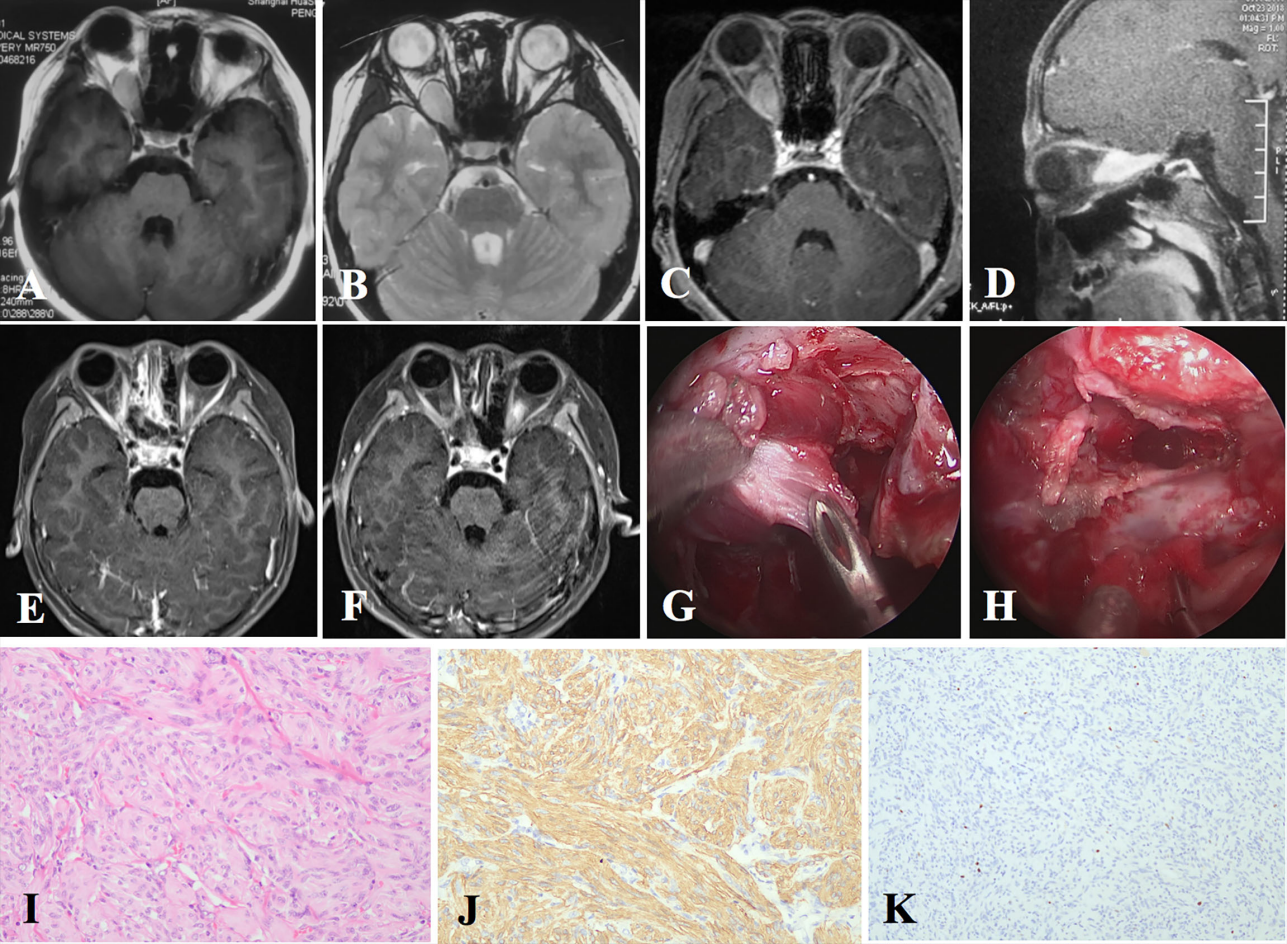
The radiological images, intraoperative pictures, and pathological staining of Case 1. **(A–D)** Preoperative MRI indicates an intraconal lesion with both medial and lateral extension to the optic nerve. **(E)** Postoperative MRI shows total resection of the tumor. **(F)** Six-month follow-up MRI shows no recurrence. **(G)** Intraoperatively, the intraconal tumor is found to be tenacious. **(H)** The tumor is totally resected. **(I)** Hematoxylin–eosin (H&E) staining shows the optic nerve completely replaced by neoplastic spindle cells with a nested pattern in microscope (original magnification ×200). **(J)** Tumor cells are positive for glial fibrillary acidic protein (GFAP) in immunochemistry (original magnification ×200). **(K)** Ki-67 index is relatively low (original magnification ×100).

An 8-year-old girl presented with heterotropia of the right eye for 1 year, and aggravated visual loss for 2 months. Neurologically, she was alert, with normal visual acuity on the left and no light perception on the right, right pupil diameter 5 mm, direct light reflex (-), indirect light reflex (+), right eye proptosis and right papilledema, otherwise intact. Preoperative computed tomography (CT) scan revealed right intra-orbital lesion, with both medial and lateral invasion to the optic nerve and right optic canal enlargement. MRI indicated that the tumor was iso-intense in T1- and T2-weighed images, homogenously enhanced on gadolinium, with a length of 27 mm on its largest axis. Right intra-orbital glioma or schwannoma was suspected preoperatively. A purely endoscopic tumor resection *via* the trans-nasal trans-orbital approach was performed. During the surgery, the medial orbital wall was removed, the tumor was resected between the interspace of the medial and inferior rectus muscles. The tumor originated from the optic nerve (see **Supplementary Video 1**). Postoperative CT and MRI scan showed total resection of the tumor. The pathology confirmed the diagnosis of pilocytic astrocytoma. The patient recovered well postoperatively and received continuous lumber drainage for 1 week (**Figure 2**).

#### 3.5.2 Case 2

**Figure 3:**
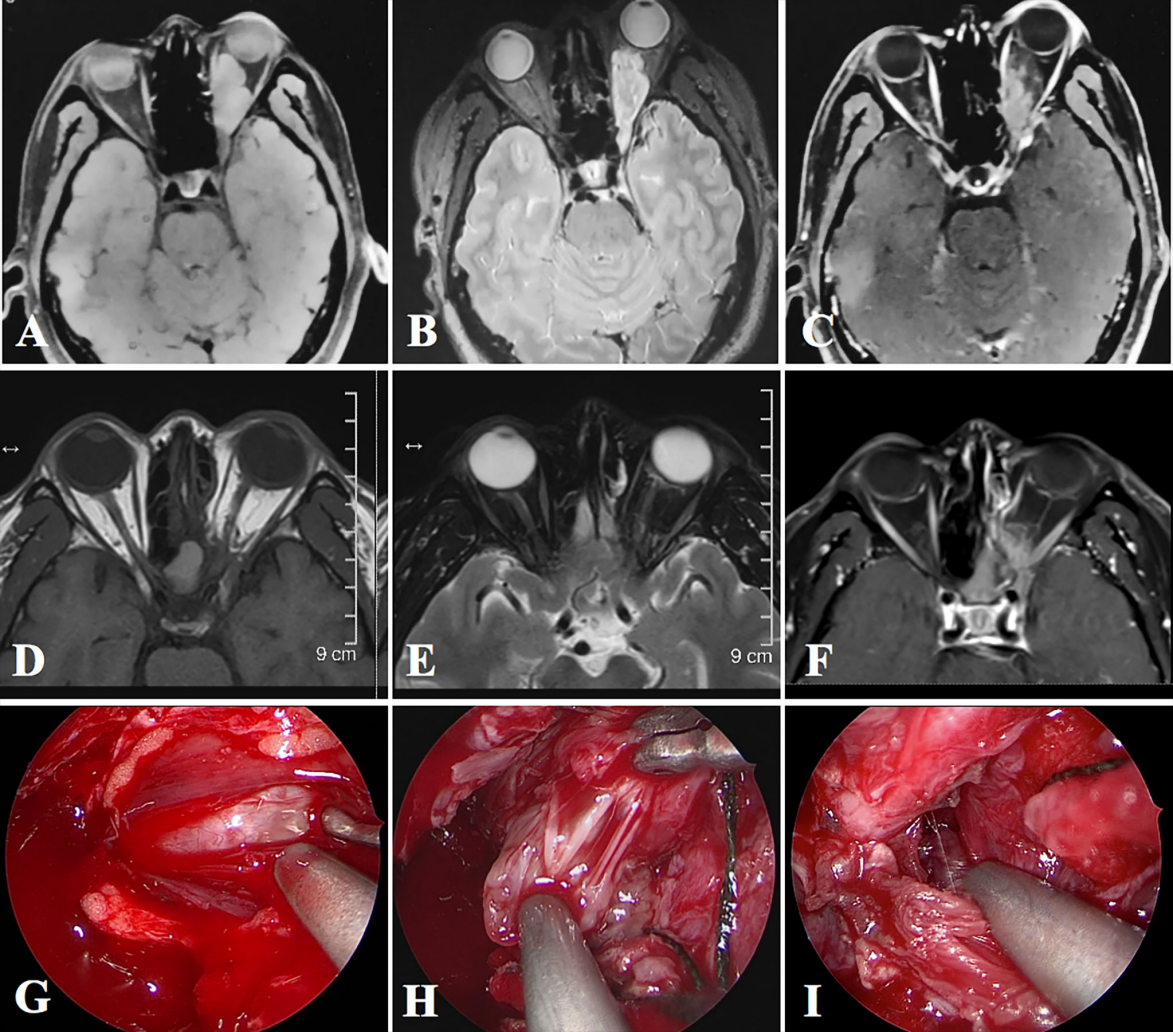
Images of Case 2. **(A–C)** Preoperative MRI shows an intraconal lesion in the left orbit, with both medial and lateral extension. **(D–F)** Six-month follow-up MRI shows no recurrence of the tumor. **(G)** Exposure of the intraconal tumor. **(H)** The carrier nerve of the tumor. **(I)** Inspection after total resection of the tumor.

A 41-year-old male complained of left eye proptosis for 6 months. Physical examination showed visual acuity 20/20 on the left, and 20/25 on the right; left eye proptosis and left corneal reflex decreased. MRI showed left intraorbital tumor with both medial and lateral extension to the optic nerve. Endoscopic endonasal tumor resection was performed, and the intraconal tumor was resected through the corridor between inferior and medial rectus muscles. The tumor originated from the branch of the oculomotor nerve, and total resection was achieved. The pathology indicated schwannoma. Postoperatively, the patient had left transient oculomotor nerve palsy, which was resolved at the 6-month follow-up (**Figure 3**).

## 4 Discussion

Intraorbital tumor is a variety of conditions that may need surgical resection. However, the surgical approach to the orbit is still controversial. External approaches and transcranial approaches have been used in the resection of intraorbital tumors, especially for those located superior or anterior to the orbit. However, the endoscopic endonasal approach provides a corridor for minimal invasive surgery of the skull base tumor, which has been developed over the last decade.

The key anatomic landmark between intraconal and extraconal orbital tumors is the position relative to the boundaries of the extraocular muscles (5). In previous reports, the extraconal tumor can be easily approached by the endonasal corridor. However, the intraconal tumors located in the superior and lateral aspect of the orbit are more difficult to be approached by this technique. McKinney and his colleagues stated that the benign soft-tissue tumors in the medial-inferior quadrant of the orbit were the most feasible for the endoscopic endonasal approach. They advocated the rule of avoiding crossing the optic nerve (5). As for the intraconal lesions, some surgeons stated that the surgical indication and approaches should be carefully considered with a wider opening of the periorbital window, and gross total resection was more difficult to achieve compared to extraconal lesions *via* this approach (7, 8). Montano summarized 70 cases of intraorbital tumors who underwent tumor resection; they recommended the endoscopic endonasal approach for primary orbital tumors located in the medial or inferior orbital walls without extra-orbital extension, the trans-eyelid approach for tumors in the upper and upper-lateral quadrants extraconally located, and the fronto-orbital approach for intraconally located tumors involving more than one quadrant (2). Rassi et al. advocated a novel CHEER staging system for evaluating the surgical approaches of intraorbital CH (12).

Some surgeons summarized a 360° “round-the-clock” surgical access to the orbit (9, 13), and they suggested the endoscopic endonasal approach for lesions at the mid or posterior orbit or orbital apex from 1 to 7 o’clock. In our case series, four of the six tumors occupied the lateral quadrants to the optic nerve, three of which were intraconal tumors. Only one case with meningioma was partially resected because of the fibrous tumor and extreme adherence with the optic nerve. The other three tumors with lateral invasion achieved gross total resection, including one pilocytic astrocytoma, one schwannoma, and one hemangioma. Postoperatively neurological deficits were unremarkable. From our perspective, the orbital tumor with lateral extension to the optic nerve is not a contraindication for the endoscopic endonasal approach.

Regarding the outcome, preservation of the cranial nerve is the top priority for the surgery of intraorbital tumor. Cranial nerves should be monitored during the surgery (14). Optic damages are often caused by direct damage, inappropriate traction, or vascular supply impairment during the dissection. Neuro-navigation is recommended during this approach (15). In our cohort, 3 of the 4 patients with intraconal lesions presented with preoperative optic neuropathy, without improvement after the surgery. Moreover, the other patient with intraconal schwannoma developed transient CN III palsy postoperatively. This result indicates that intraconal lesions are more vulnerable for cranial nerve impairment during the surgery.

It is believed that removal of the lamina papyracea or even the medial wall of the orbit does not cause eyeball disposition. However, removal of the periorbita or additional fat dissection may result in orbital fat herniation that can lead to permanent or transient diplopia, enophthalmos, and strabismus (8). Some surgeons suggested reconstruction of the medial orbital wall with bone and nasoseptal flap in case of large defect (16, 17). It was reported to use a thick silastic sheet in the nasal cavity to prevent orbital content herniation then remove 4 weeks after the surgery (18).

Still, there are some limitations of this approach. A preoperative evaluation of the accessibility of the medial and lateral borders of the tumor should be performed. Potential malignancy of the lesion should also be considered because the endoscopic endonasal tumor resection could lead to incomplete resection (19). This approach would be challenging for fibrous tumors with lateral extension to the optic nerve. In such cases, other approaches or staged surgeries should be considered. Adjuvant therapy should also be recommended as an alternative treatment.

In conclusion, the endoscopic endonasal approach is applicable for selected intraorbital tumors both medially and laterally located. The pathology of the lesion and adhesion to adjacent neurovascular structures should also be considered to determine the most appropriate approach.

## Data Availability Statement

The raw data supporting the conclusions of this article will be made available by the authors, without undue reservation.

## Ethics Statement

The studies involving human participants were reviewed and approved by the Huashan Hospital Institutional Review Board. Written informed consent to participate in this study was provided by the participants’ legal guardian/next of kin.

## Author Contributions

Study conception and design: WZ and WH. Acquisition of data and follow-up: XZ. Surgical participation: XZ, WH, KQ, GY, ZY, XW, JP, LC, and WZ. Pathological confirmation: ZD. Drafting of manuscript: XZ and WH. Critical revision: all authors. XZ and WH contributed equally to this study and shared the first authorship. All authors contributed to the article and approved the submitted version.

## Funding

This study was supported and granted by the CAMS Innovation Fund for Medical Sciences (CIFMS, 2019-I2M-5-008).

## Conflict of Interest

The authors declare that the research was conducted in the absence of any commercial or financial relationships that could be construed as a potential conflict of interest.

The handling editor declared a shared parent affiliation with the authors at time of review.

## Publisher’s Note

All claims expressed in this article are solely those of the authors and do not necessarily represent those of their affiliated organizations, or those of the publisher, the editors and the reviewers. Any product that may be evaluated in this article, or claim that may be made by its manufacturer, is not guaranteed or endorsed by the publisher.
