# Supplementary material for: Endoscopic Endonasal Intraconal Approach for Orbital Tumor Resection: Case Series and Systematic Review
Source: Front Oncol. 2022 Jan 3;11:780551. doi: 10.3389/fonc.2021.780551 (PMC8761671; doi:10.3389/fonc.2021.780551)
Supplement: Supplementary file 1 [file DataSheet_1.docx]

**Supplementary data**

Case 3:

A 46-year-old female complained of right eye proptosis for 2 months, neurological examination showed normal visual acuity on both eyes and right eye exophthalmos. MRI indicated right intraorbital tumor, with no significant enhancing after contrast agent injection. A purely endoscopic transnasal approach was applied in the surgery. During the surgery, after the right medial orbital wall was removed, the extraconal tumor was exposed. Finally, the tumor received total resection which was confirmed by post-op MRI. Pathology confirmed the diagnosis of angioleiomyoma. The patient recovered well post-operatively, with CN VI palsy resolved (**Supplementary Figure 1**).

Case 4:

A 43-year-old female presented with right eye discomfort and right visual acuity deterioration for one year, physical examination demonstrated the visual acuity was 0.6 in the right and 1.0 in the left, with right eye proptosis. MRI indicated a right intraorbital lesion in the orbit apex, with homogenous enhancement after contrast. Endoscopic transnasal approach was applied for the patient. During the surgery, the tumor was exposed after removal of the lamina papyracea and incision of the periorbita. The tumor was extreme tenacious with abundant blood supply, the optic nerve was encased in the tumor. At this time, total tumor resection was risky to achieve, so we decided to perform the partial resection of those medial to the optic nerve. And the right optic canal bony decompression was performed. After the operation, the visual acuity of right eye was 3-meter finger-counting and proptosis was improved (**Supplementary Figure 2**).

Case 5:

A 34-year-old female complained of right visual loss for 6 months, with hand movement before eye on the right and right pupil dilated. MRI revealed a mass located in the right orbit, with heterogeneous enhancement after contrast. Endonasal endonasal interconal approach was used and total resection of the tumor was achieved (see **Supplementary Video 2**), which was confirmed by post-operative MRI. Histopathology findings indicated the diagnosis of schwannoma (**Supplementary Figure 3**).

Case 6:

A 47-year-old female complained of left visual loss for 1 month, with visual acuity of counting fingers 30 centimeters on the left eye. MRI revealed a mass located in the left orbit apex, with heterogeneous enhancement after contrast. Tumor resection was performed via endoscopic transnasal trans-orbital approach, and total resection was achieved indicated by post-operative MRI. The pathology was cavernous hemangioma, with positive CD34 and Smooth Muscle Actin (SMA) staining. The patient left visual acuity was improved immediately after the surgery (**Supplementary Figure 4**).

**Supplementary Figure Legends:**

**Supplementary Figure 1**. Images of Case 3. A-D. Preoperative MRI shows a lesion located in the extraconal of the right orbit, without obvious enhancement after gadolinium administration. E-F. Postoperative MRI confirms gross total resection of the tumor. G. Exposure of the lateral wall of the right orbit. H. En bloc removal of extraconal lesion. I. H&E staining shows the tumor composed of juxtaposed vascular channels with muscularis (original magnification ×100). J. Endothelial cells are positive for CD34 (original magnification ×100). K. Their channel muscularis is positive for SMA (original magnification ×100).

**Supplementary Figure 2**. Images of Case 4. A-C. Preoperative MRI shows an intraconal lesion in the right orbit, with the optic nerve encased. D-E. MRI 3 months after surgery shows tumor stable. F. Intra-operatively, the medial part of the tumor is removed.

**Supplementary Figure 3**. Images of Case 5. A-C. Preoperative MRI indicates a right orbital intraconal tumor, with heterogeneous enhancement. D. Three-month post-operative MRI shows the total resection of the tumor. E. Exposure of the lateral wall of right orbit. F. Exposure of the intraconal tumor after dissection of the medial rectus muscle. G. En bloc resection of the tumor (asterisk) is performed.

**Supplementary Figure 4**. Images of Case 6. A-C. Axial preoperative MRI shows left orbital lesion, with heterogeneous enhancement, protruding into the cavernous sinus. D. Exposure of the lateral wall of left orbit. E. Dissection of the medial rectus muscle. F. Exposure of the tumor (asterisk). G. H&E staining shows a mass lesion consisted with large cystically dilated vessels surrounded by fibrous capsules (original magnification×100). H. Positive CD34 staining marks the vascular endothelial cells (original magnification×100). I. Postoperative MRI confirms total resection of the lesion.

**Video illustration:**

**Supplementary Video 1**: This video illustrates a child with right intra-orbital pilocytic astrocytoma (Case 1), who underwent purely endoscopic endonasal interconal tumor resection. After the sphenoidotomy, sellar floor, right optic canal and lamina papyracea were open. The Doppler probe and navigation were applied to localize the carotid and the tumor in the orbit. The periorbita was cut open and orbital fat was exposed. Then the medial rectus muscle and inferior rectus muscle were retracted, and the fibrous tumor with white capsule was exposed. The tumor was debulked by piecemeal fashion using Cavitron ultrasonic surgical aspirator (CUSA). Once the tumor was detached from the orbital content, the remnant in the optic canal was removed. CSF leakage from the optic canal was noticed. At last, the tumor was removed en bloc. Optic canal decompression was performed. The skull base was reconstructed using the fat graft and nasoseptal flap harvested at the end of the surgery.

**Supplementary Video 2**: This video shows the surgical procedure of Case 5 with a right intraconal schwannoma. After lamina papyracea of right orbit was removed, periorbita was cut open with a blade. Orbital fat tissue was exposed, and medial rectus muscle and inferior rectus muscle were retracted. Thereafter, the intraconal whitish tumor was identified. The tumor was dissected and en bloc resected carefully. No CSF leakage was noticed.
