# Supplementary figures and images for: Endoscopic Endonasal Intraconal Approach for Orbital Tumor Resection: Case Series and Systematic Review
Source: Front Oncol. 2022 Jan 3;11:780551. doi: 10.3389/fonc.2021.780551 (PMC8761671; doi:10.3389/fonc.2021.780551)

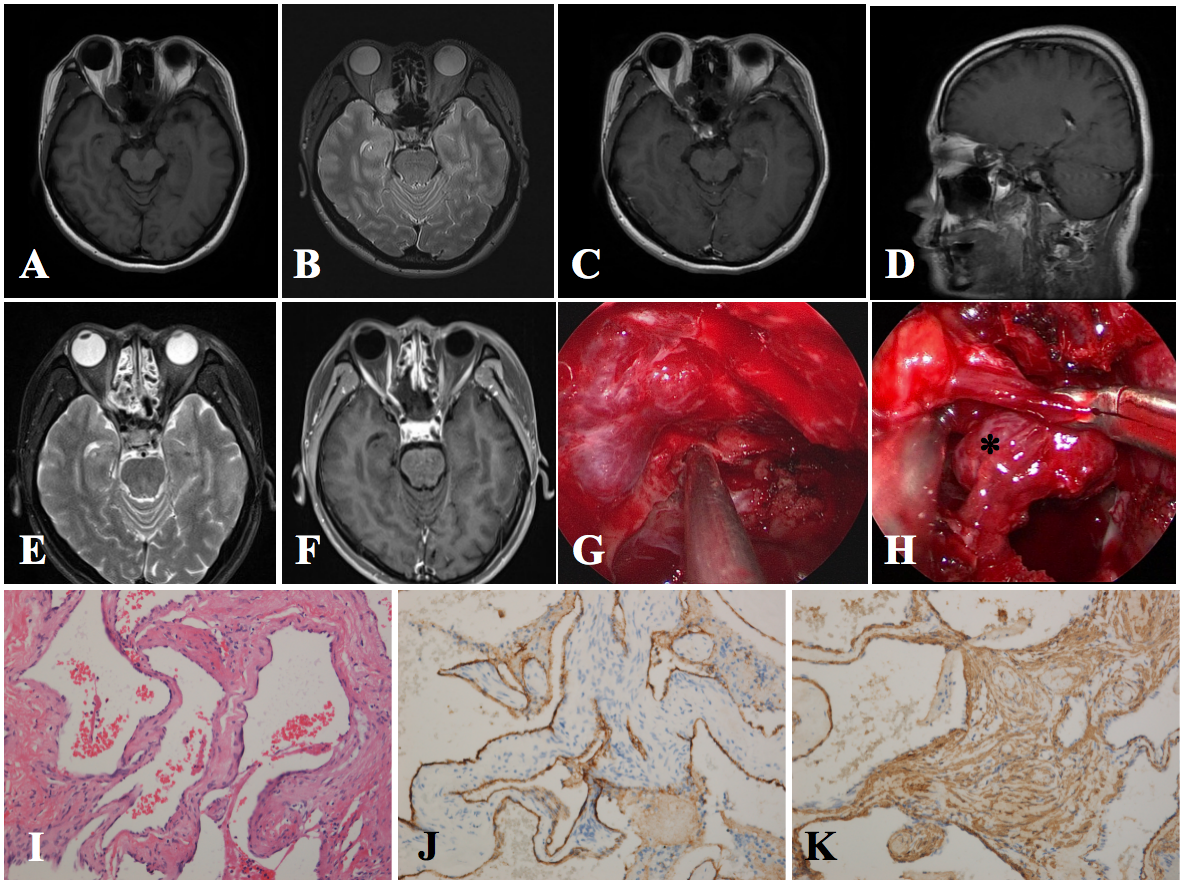

Supplement: Supplementary file 2 [file Image_1.tif]

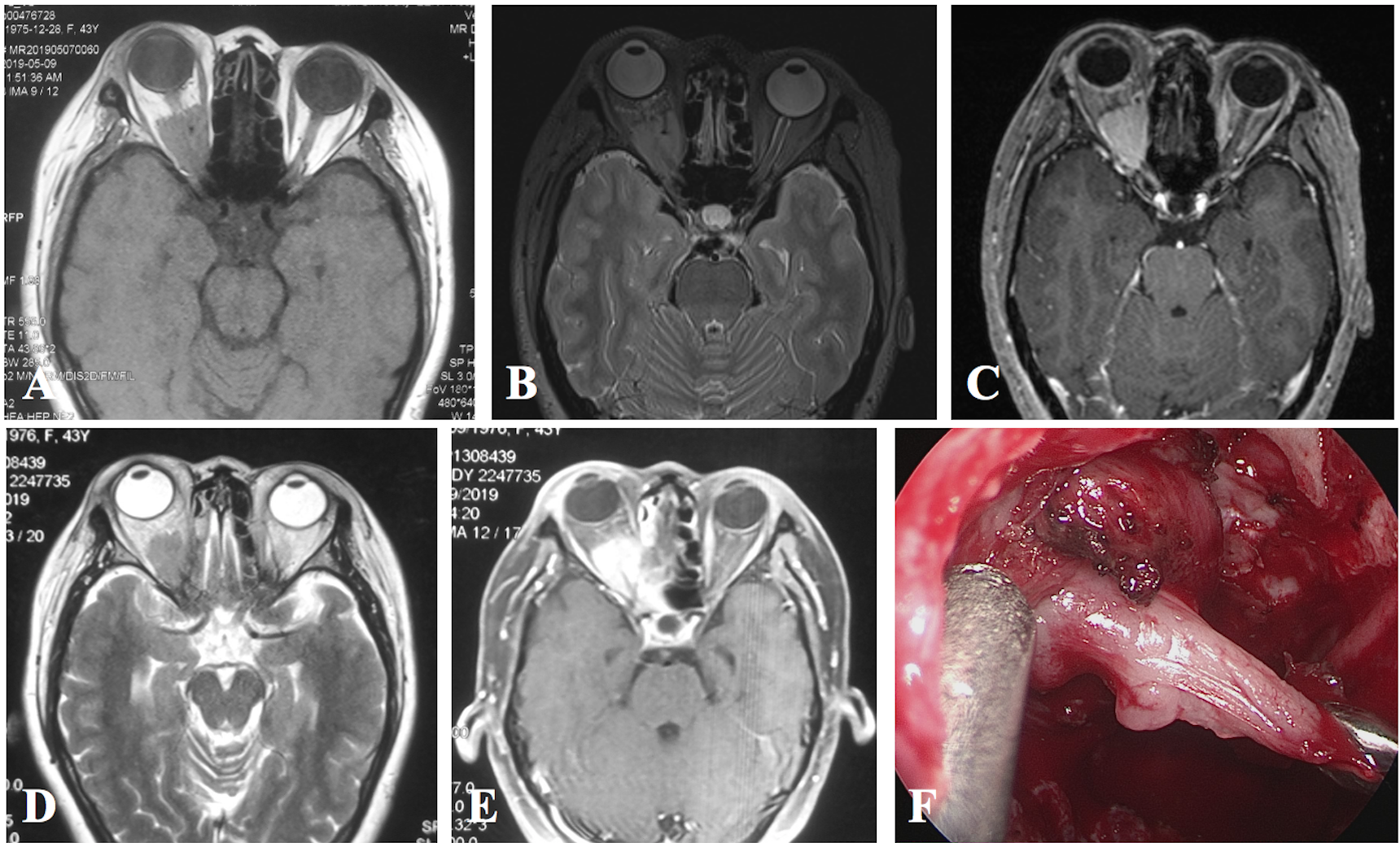

Supplement: Supplementary file 3 [file Image_2.tif]

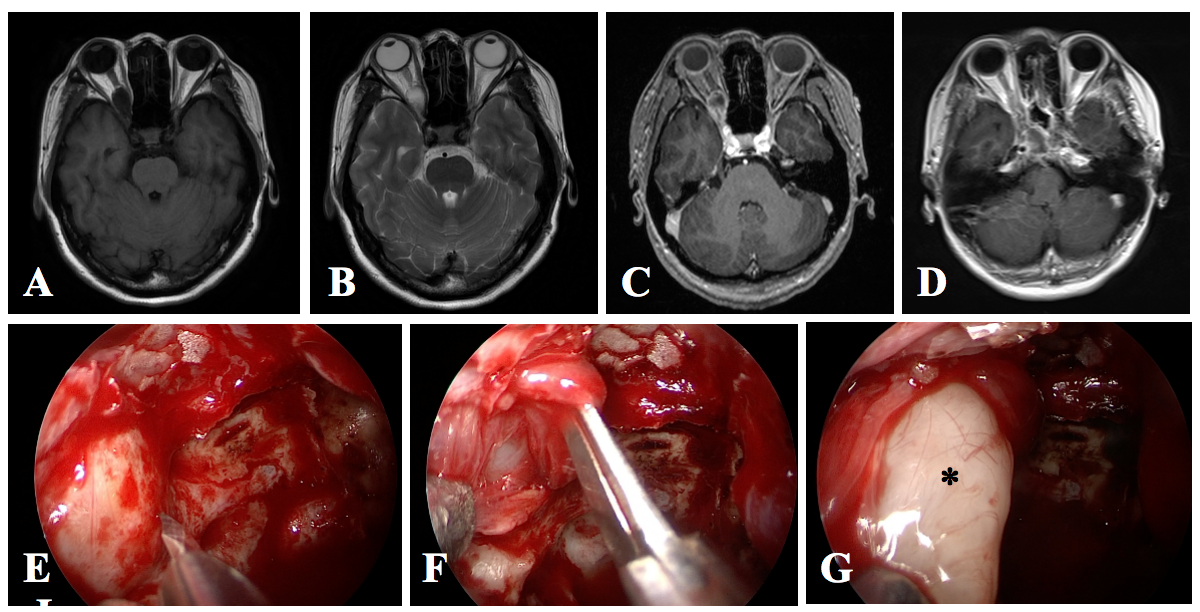

Supplement: Supplementary file 4 [file Image_3.tif]

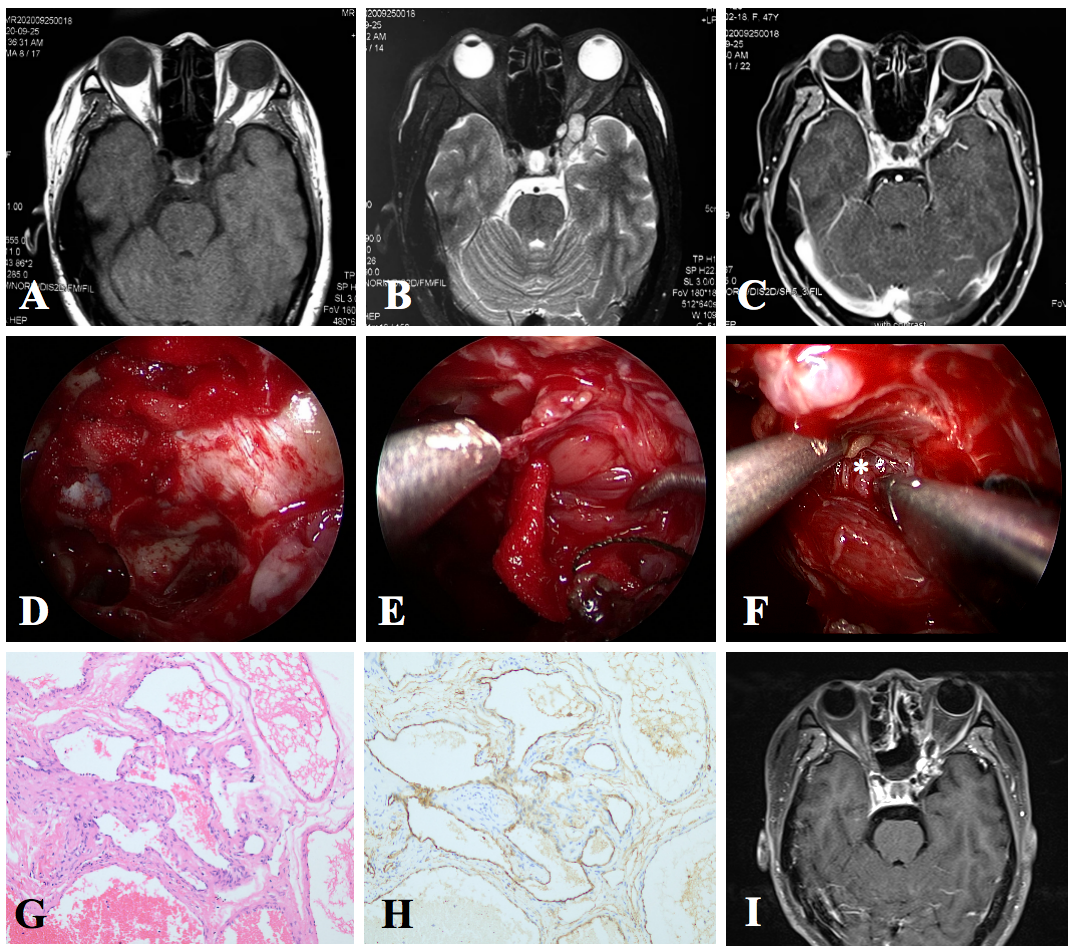

Supplement: Supplementary file 5 [file Image_4.tif]
